# Risk Factors for Mediastinal Lymph-Node Metastasis in Siewert Type I/II Esophagogastric Junction Cancers

**DOI:** 10.3390/cancers18132180

**Published:** 2026-07-07

**Authors:** Yoshiaki Shoji, Kazuo Koyanagi, Miho Yamamoto, Akihito Kazuno, Yamato Ninomiya, Kohei Kanamori, Takatoshi Seki, Kohei Tajima, Rie Nakashima, Masaki Mori

**Affiliations:** Department of Gastroenterological Surgery, Tokai University School of Medicine, 143 Shimokasuya, Isehara 259-1193, Kanagawa, Japan; y.shoji@tokai.ac.jp (Y.S.); miho-n@tokai.ac.jp (M.Y.); tokaikazuno@tokai.ac.jp (A.K.); y.ninomiya@tokai.ac.jp (Y.N.); k.kanamori@tokai.ac.jp (K.K.); st9054@tokai.ac.jp (T.S.); xrw6270@tokai.ac.jp (K.T.); nr3419@tokai.ac.jp (R.N.); mori.masaki.r@tokai.ac.jp (M.M.)

**Keywords:** esophagogastric junction cancer, adenocarcinoma, Siewert type, mediastinal lymph-node metastasis, risk factor

## Abstract

Background/Objectives: Currently, a definitive diagnostic strategy for predicting mediastinal lymph-node (MLN) metastasis remains poorly established in Siewert Type I and II esophagogastric junction adenocarcinoma (EGJAC). This study aimed to evaluate long-term survival and identify objective, reliable preoperative risk factors for MLN metastasis to optimize perioperative treatment and surgical approach. Methods: We retrospectively reviewed a well-defined cohort of 133 patients with Siewert Type I (33.1%) and II (66.9%) EGJAC who underwent R0 resection and complete follow-up at our institution between 2005 and 2025. Preoperative risk factors for histological MLN metastasis were evaluated using receiver operating characteristic (ROC) curve analysis and univariate/multivariate logistic regression models. Results: Thorough upper, middle, and lower MLND were completed in 57.9%, 72.9%, and 94.7% of patients, respectively. Pathological MLN metastasis was confirmed in 31.6% of patients and was significantly associated with poor overall and recurrence-free survival. ROC analysis demonstrated that an MLN short-axis diameter of ≥5 mm on CT, esophageal involvement length (EIL) in upper GI series ≥ 30 mm, and EIL in endoscopy ≥ 60 mm predict histological MLN metastasis. In univariate analysis, Siewert Type I, EIL in upper GI series ≥30 mm, EIL in endoscopy ≥ 60 mm, clinical tumor depth ≥ cT3, and CT node size ≥ 5 mm were correlated with MLN metastasis. Multivariate logistic regression analysis revealed that a clinical tumor depth of ≥cT3, EIL in upper GI series ≥ 30 mm, and a CT node size of ≥5 mm were independent predictors of MLN metastasis. Conclusions: Clinical tumor depth, EIL, and size of MLN are powerful independent preoperative risk factors for MLN metastasis in Siewert Type I/II EGJAC. Utilizing these objective indices may help identify high-risk patients requiring intensive perioperative multidisciplinary strategies.

## Simple Summary

Choosing the optimal clinical approach for Siewert Type I and II esophagogastric junction adenocarcinoma (EGJAC) remains challenging due to the high risk of mediastinal lymph-node (MLN) metastasis. Patients with positive MLN have a poor prognosis, necessitating more intensive perioperative treatment. Furthermore, while extensive MLN dissection (MLND) may be required for these patients, it also carries the risk of inducing severe postoperative complications, such as recurrent laryngeal nerve paralysis. Therefore, predicting MLN metastasis and establishing individualized treatment strategies are of paramount importance. In this retrospective study of 133 patients with Siewert Type I/II EGJAC, we investigated objective preoperative indicators to accurately predict MLN involvement. Our findings demonstrated that estimated tumor depth and MLN size on CT are powerful, independent predictors of histological MLN metastasis, in addition to esophageal involvement length. Incorporating these objective criteria into initial staging algorithms may assist in identifying high-risk candidates who truly require intensive perioperative treatment and extended transthoracic MLND, thereby optimizing personalized multimodal treatment strategies while mitigating unnecessary surgical invasiveness.

## 1. Introduction

Incidence rates for esophagogastric junction adenocarcinoma (EGJAC) are rising rapidly worldwide, possibly due to increased body weight, increasing gastroesophageal reflux disease, and decreasing levels of chronic infection with H. pylori [1,2]. Although there is still room for debate, perioperative chemotherapy with surgery has been regarded as the standard of care for locally advanced EGJAC in Western countries [3,4,5], whereas surgery with or without adjuvant chemotherapy has been widely accepted in Japan, since the significance of preoperative therapy has not yet been established [6].

The Siewert classification is globally used to categorize EGJAC, based on the location of the tumor epicenter. Resectable Siewert Type III cancers, located 2 to 5 cm below the EGJ, are usually treated as upper stomach cancer [7,8]. Until recently, treatment strategies for EGJAC with esophageal invasion, often classified as Siewert Type I (located 5 to 1 cm above the EGJ) or Type II (located 1 cm above to 2 cm below the EGJ) tumors, have not been determined, due to the potential for mediastinal lymph-node (MLN) metastasis. Patients with positive MLN have a poor prognosis and may require more intensive perioperative therapy and/or extensive MLN dissection (MLND). On the contrary, total MLND may lead to recurrent laryngeal nerve (RLN) palsy, which significantly affects the quality of life and prognosis of the patients [9,10], and should be avoided when unnecessary. Therefore, accurate diagnosis of MLN metastasis and the therapeutic efficacy of MLND remain critical clinical challenges that continue to be a subject of ongoing debate.

In 2017, Yamashita et al. conducted a questionnaire-based nationwide retrospective study in Japan to define the optimal extent of LND for EGJ cancers [11]. In this study, EGJ cancer was defined as having its tumor epicenter within 2 cm above or below the EGJ, regardless of the histological type, according to the definition promulgated by the Japanese Gastric Cancer Association [12] and the Japanese Esophageal Society [13], and patients with tumors ≤ 4 cm were considered eligible. In total, 1560 patients with Siewert Type I/II EGJAC were enrolled. The estimated survival benefit by LND was high for the abdominal LN along the lesser curvature, right and left cardia, and the left gastric artery; however, the optimal extent of MLND could not be determined due to the low rate of upper/middle MLND, which was below 20%.

To clarify the frequency of MLN metastasis according to esophageal involvement length (EIL), Kurokawa et al. reported the results of a prospective multicenter study in 2021 [14], in which patients with cT2–T4 cancers located within 2 cm of the EGJ, including esophageal squamous cell carcinoma (ESCC), were enrolled. Upper MLND was performed for patients with EIL > 3 cm, whereas patients with EIL ≤ 3 cm underwent lower MLND only. For the 98 patients who had EIL > 3 cm (67 EGJAC and 31 ESCC), the rate of right RLN LN metastasis was 10.7% (2/28) when the EIL exceeded 4 cm. The final analysis, which evaluated the therapeutic efficacy index (TEI) for each LN station [15], concluded that total MLND should be considered for patients with EIL ≥ 3 cm; however, the effectiveness of upper MLND was limited in patients after neoadjuvant treatment.

As a result of these current studies, a recommended algorithm for a standardized LND and surgical approach was proposed, in which the extent of MLND should be tailored to the EIL. However, some issues remain unsolved. First, there is a limited number of studies and cases, especially for those with EGJAD who underwent total MLND. Second, it is difficult to accurately measure the distance from the EGJ to the tumor epicenter and proximal margin in patients with large tumors, circumferential tumors, and hiatal hernia. Third, there is a limited accuracy of preoperative measures when detecting nodal involvement, as has been discussed elsewhere [16,17,18]. And finally, EGJAC with long EIL, usually classified as Siewert Type I, was often excluded in previous studies.

The aim of this study was to identify independent and objective factors to predict MLN metastasis in EGJAC with esophageal involvement. These factors may enable the selection of an optimal treatment strategy to improve prognosis and minimize surgical invasion for individual patients with EGJAC.

## 2. Materials and Methods

### 2.1. Patients

Patients who underwent R0 resection for histologically confirmed Siewert Type I or II EGJAC at Tokai University Hospital from January 2005 to December 2025 were selected from a prospectively maintained database. Five patients who underwent non-curative resection and one patient with synchronous mediastinal germ cell tumor were excluded from the study. After 2019, patients with advanced EGJAC, i.e., tolerable to chemotherapy, underwent neoadjuvant chemotherapy followed by surgical treatment and were included in the study. Minimally invasive surgery, including robot-assisted surgery, was introduced at our institution in 2011, and patients who underwent these procedures were also included in this study. Eligible patients with pStage II and pStage III diseases underwent adjuvant S-1 monotherapy [19] and Docetaxel plus S-1 therapy [20], respectively, and were also included.

### 2.2. Surgical Procedure

**Figure 1:**
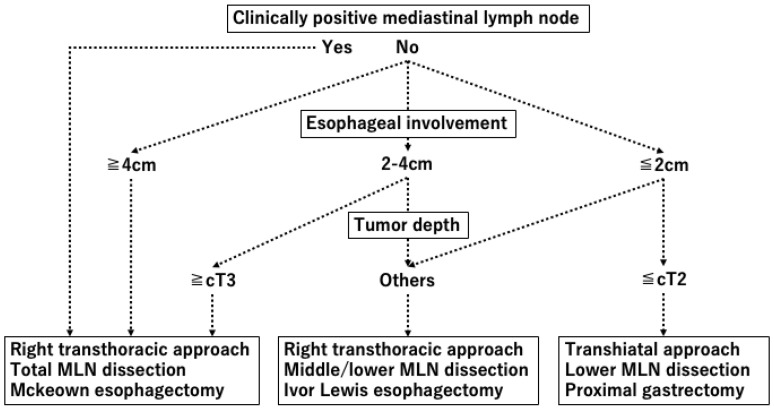
Surgical algorithm for Siewert Type I/II esophagogastric cancer at our institute. Surgical approach and the extent of MLND were decided based on esophageal involvement and the estimated tumor depth.

The surgical algorithm for Siewert Type I and II EGJAC at our institute is shown in Figure 1. Surgical approach and the extent of MLND were decided based on EIL and the estimated tumor depth. Briefly, patients with EIL ≤ 2 cm and tumor depth ≤ cT2 underwent proximal gastrectomy (with inferior esophagectomy) and lower mediastinal LND [18,21,22]. Other patients underwent transthoracic esophagectomy as previously described [23]. For patients with clinically positive MLN and/or 4 cm or longer EIL, McKeown esophagectomy with total MLND was performed [24]. Other patients underwent Ivor Lewis esophagectomy with middle and lower MLND as previously described [25]. Patients with large tumors invading the lower stomach underwent total gastrectomy (with inferior esophagectomy) and were also included in the study.

### 2.3. Image Analysis

Endoscopic examination, upper gastrointestinal series (UGI), CT, and PET-CT were performed at the initial visit. Endoscopy and CT were also performed 4 weeks after the final administration of neoadjuvant treatment for eligible patients. Clinical and pathological stages were classified according to the Union for International Cancer Control (UICC) TNM staging system for EGJ cancer [26]. Lymph-node station numbers were defined according to the Japanese Classification of Gastric Carcinoma, 3rd English edition [12], and the Japanese Classification of Esophageal Cancer, 12th edition [27].

**Figure 2:**
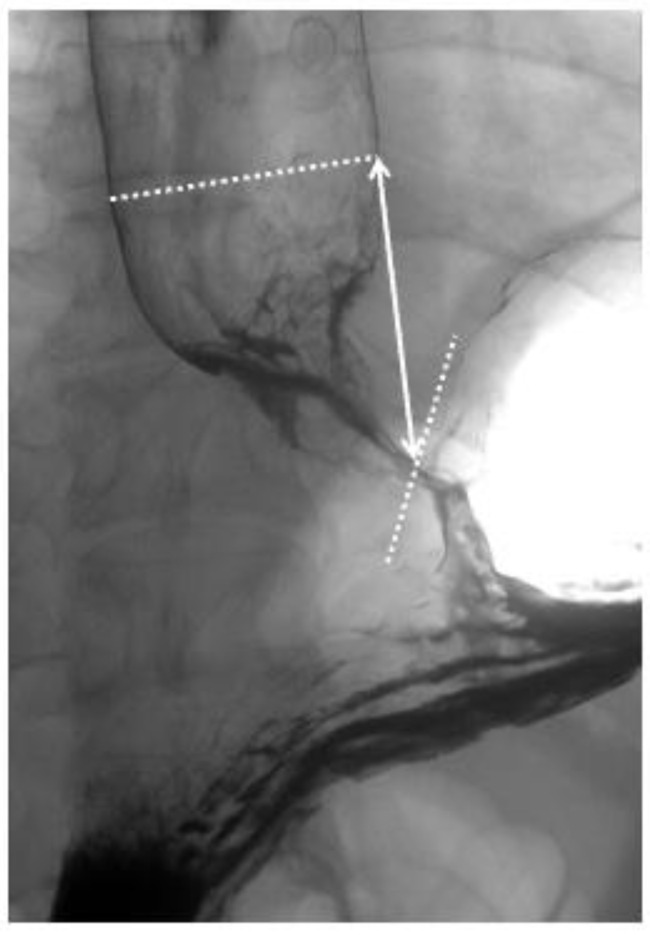
Esophageal involvement length measured by upper GI series (UGI EIL). The straight-line distance from the upper margin of the tumor to the His angle was defined as UGI EIL in this study.

EIL was measured using both endoscopy and UGI. Endoscopic examinations were either performed or supervised by a Board-Certified Trainer of the Japan Gastroenterological Endoscopy Society, and the EIL (endoscopic EIL) was measured. Data for endoscopic EIL was collected retrospectively based on endoscopy reports, which yielded a total of 86 patients. During UGI, the straight-line distance from the upper margin of the tumor to the His angle (UGI EIL) was measured and recorded using double-contrast imaging in the upright position (Figure 2).

The shorter diameter of all visible MLNs was measured and recorded to analyze the correlation between MLN size and metastasis. On PET-CT, MLNs were defined as metastatic if they exhibited fluorodeoxyglucose (FDG) accumulation higher than the physiological background levels. Because it is not covered by national health insurance for early-stage cancer in Japan, PET-CT was performed only when advanced cancer was suspected, resulting in 99 cases being evaluated.

### 2.4. Statistical Analysis

Clinicopathological outcomes, overall survival (OS), and recurrence-free survival (RFS) were evaluated. OS/RFS was calculated from the date of surgery to the date of death/recurrence. Statistical analyses were performed using Python (version 3.10). OS and RFS were compared using the log-rank test. Continuous variables were compared using the Mann–Whitney U test or Student’s *t*-test, and categorical variables were analyzed using the chi-square test or Fisher’s exact test. To determine the optimal cut-off values of EIL and size of MLN for MLN metastasis, receiver operating characteristic (ROC) curve analysis was conducted. The cut-off points were identified based on the Youden Index (maximum [sensitivity + specificity − 1]). To identify preoperative risk factors associated with MLN metastasis, univariate and multivariate logistic regression analyses were performed. Variables with a *p*-value < 0.05 in the univariate analysis were entered into the multivariate logistic regression model. Odds ratios (ORs) and 95% confidence intervals (CIs) were calculated for each factor. To account for potential sparse-data bias and ensure model stability, Firth’s penalized likelihood logistic regression was performed for both univariate and multivariate analyses. All statistical tests were two-sided, and a *p*-value < 0.05 was considered statistically significant. Unless otherwise indicated, data are presented as the median and range.

## 3. Results

### 3.1. Patient Backgrounds

The study cohort included 133 patients with EGJAC who underwent R0 resection from 2005 to 2025 at our institute. In total, 44 (33.1%) patients had Siewert Type I tumors, and the EIL ranged from 0 to 114 mm. Furthermore, 95 (71.4%) patients had advanced tumors classified as cStage II or above. Of them, 26 (19.5%) patients underwent neoadjuvant treatment (Table 1).

### 3.2. Surgical and Pathological Outcomes

Upper, middle, and lower MLNDs were performed for 77 (57.9%), 97 (72.9%), and 126 (94.7%) patients, respectively. Notably, 30 (22.6%) patients developed postoperative complications, where anastomotic leakage (18, 13.5%) was most frequently observed. Others included one case each of intestinal necrosis, pancreatic fistula, and small bowel obstruction. Pathologically, 87 (65.5%) patients had pStage II or higher advanced diseases. MLN metastasis was observed in 42 (31.6%) patients (Table 2).

Details of MLN metastasis are shown in Table 3. Lymph-node stations with metastatic rates >10% include stations #106recL, #106recR, #108, #110, and #112.

### 3.3. Survival Outcomes

**Figure 3:**
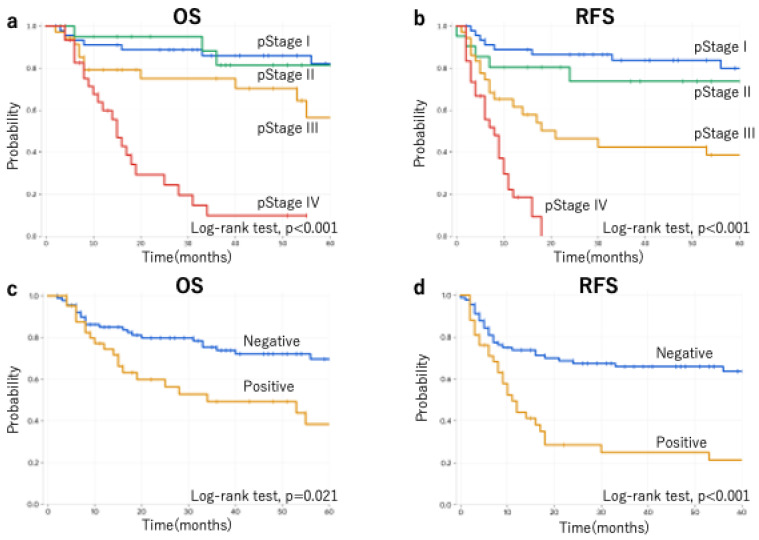
Overall survival (OS) and recurrence-free survival stratified by pStage (**a**,**b**) and presence or absence of mediastinal lymph-node metastasis (**c**,**d**).

Results of the survival analysis are shown in Figure 3. Significant differences in OS and RFS were observed based on pStage (Figure 3a,b) and the presence or absence of MLN metastasis (Figure 3c,d).

### 3.4. Risk Factors for Mediastinal Lymph-Node Metastasis

#### 3.4.1. Esophageal Involvement Length

**Figure 4:**
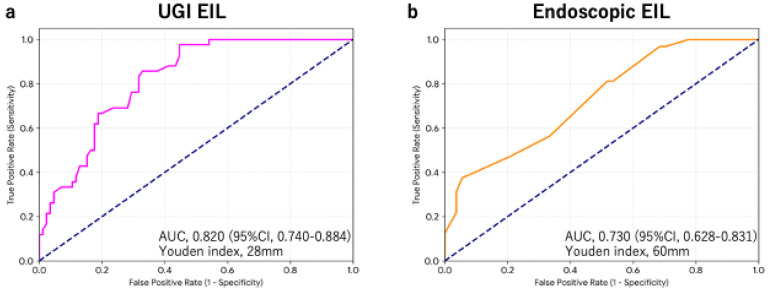
ROC analysis regarding esophageal involvement length and mediastinal lymph-node metastasis evaluated by (**a**) upper GI series and (**b**) upper gastrointestinal endoscopy.

UGI and endoscopic EILs were evaluable in 127 and 86 patients, respectively. Cut-off values of UGI EIL and endoscopic EIL for MLN metastasis utilizing the Youden Index were 28 mm (sensitivity, 97.6%; specificity, 55.3%) and 60 mm (sensitivity, 37.5%; specificity, 94.4%), respectively (Figure 4).

#### 3.4.2. Size of Mediastinal Lymph Nodes and Metastasis

**Figure 5:**
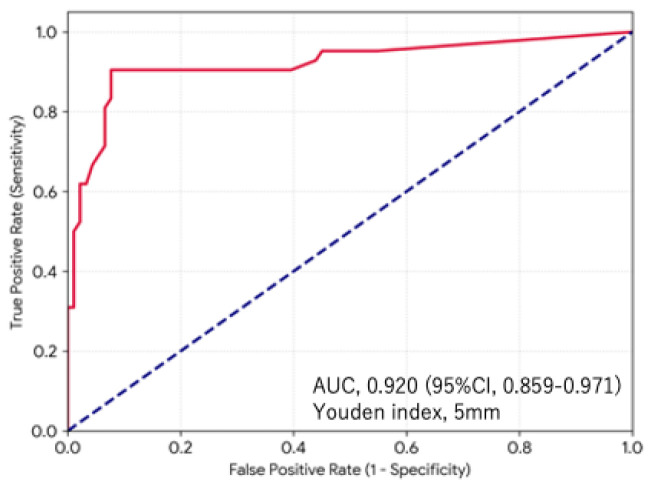
ROC analysis regarding the size of mediastinal lymph-node metastasis evaluated by CT images.

The association between the size of MLN in CT images and MLN metastasis was analyzed in all 133 patients (Figure 5). The sensitivity, specificity, positive predictive value, negative predictive value, and diagnostic accuracy of MLN size ≥ 5 mm on preoperative CT images for MLN metastasis were 90.5%, 92.3%, 84.4%, 95.5%, and 91.7%, respectively (Youden Index, 5 mm).

#### 3.4.3. Risk Factors for Mediastinal Lymph-Node Metastasis

Univariate and multivariate analyses were performed to identify predictors of MLN metastasis, incorporating the cut-off values for UGI EIL, endoscopic EIL, and MLN size determined by ROC curve analysis. In the univariate analysis, the incidence of MLN metastasis was significantly higher in cases with Siewert Type I tumors (*p* = 0.006), endoscopic EIL ≥ 60 mm (*p* < 0.001), UGI EIL ≥ 30 mm (*p* = 0.001), cT3 or deeper tumor invasion (*p* < 0.001), MLN size ≥ 5 mm (*p* < 0.001), and positive FDG uptake in MLN on PET-CT (*p* = 0.001, Table 4). Subsequently, significant factors were evaluated in the multivariate analysis. However, to ensure a more accurate multivariate analysis, endoscopic EIL ≥ 60 mm and positive FDG uptake in MLN on PET-CT were excluded from the model due to their small sample sizes. The multivariate analysis revealed that UGI EIL ≥ 30 mm (*p* = 0.008), cT3 or deeper tumor invasion (*p* = 0.043), and MLN size ≥ 5 mm (*p* < 0.001) were independent predictors of MLN metastasis (Table 5).

Further univariate analysis was performed to identify risk factors for MLN metastasis in the subgroup of patients with Siewert Type I tumors (Table 6). The incidence of MLN was significantly higher in cases with endoscopic EIL ≥ 60 mm (*p* = 0.01), cT3 or deeper tumor invasion (*p* < 0.001), MLN size ≥ 5 mm (*p* < 0.001), and positive FDG uptake in MLN on PET-CT (*p* = 0.01).

Finally, we stratified the risk of MLN metastasis by creating a 2 × 2 contingency table based on two significant predictors: cT and UGI EIL (Table 7). Patients satisfying both criteria showed a high MLN metastasis rate (62.0%), whereas no MLN metastasis was detected in the double-negative group. Of note, all patients who had MLN metastasis in the single-positive group had MLN ≥ 5 mm, whereas four patients with MLN metastasis in the double-positive group had MLN < 5 mm.

## 4. Discussion

In the present study, we evaluated the preoperative risk factors for MLN metastasis in a well-defined cohort of 133 patients with Siewert Type I/II EGJAC who underwent R0 resection and were followed up with entirely at our institution. Our survival analysis demonstrated that both advanced pStage and the presence of MLN metastasis were significantly associated with poor OS/RFS, highlighting the profound prognostic impact of mediastinal nodal involvement. Furthermore, through objective assessments, multivariate logistic regression analysis revealed that UGI EIL ≥ 30 mm, clinical tumor depth of cT3 or deeper, and a preoperative MLN short-axis diameter of 5 mm or larger on CT were independent predictors of MLN metastasis. These findings offer crucial, objective indices that can optimize treatment strategies and tailor multidisciplinary treatment algorithms for individual patients with EGJAC.

The clinical classification and optimal management of Siewert Type I/II tumors remain a subject of ongoing debate worldwide. In daily clinical practice, accurately determining the precise tumor center—and thus strictly differentiating between Siewert Type I and Type II—is frequently challenging, particularly in patients presenting with circumferential tumors or coexisting esophageal hiatal hernias. Further, our findings suggest that EIL possesses greater predictive value for MLN metastasis compared to the Siewert classification. Therefore, analyzing Siewert Type I and II tumors (tumors with esophageal involvement) together as a single, continuous entity, as done in this study, carries profound clinical relevance for real-world surgical oncology. In fact, a previously conducted multicenter study [14] included a limited number of Siewert Type I tumors (with an EIL of 1–2 cm) as eligible cases, whereas the vast majority of cases were excluded. A major strength of our study is the inclusion of a substantial number of Siewert Type I tumors (33.1%) within a large single-center cohort of 133 cases. Reflecting our proactive surgical policy for these junctional lesions, extensive MLND was performed at an exceptionally high rate: complete MLND was completed in 57.9% of patients, and middle MLND in 72.9%. This high rate of thorough dissection ensures the accuracy of our pathological nodal staging and minimizes the risk of underdiagnosing occult MLN metastasis.

The likelihood of MLN metastasis largely depends on EIL. According to a pivotal multi-institutional prospective study conducted in Japan, the incidence of MLN metastasis correlates strongly with the longitudinal extent of the disease, and extensive LND up to the upper mediastinum is strongly required when the esophageal invasion length exceeds 4 cm [14]. Furthermore, a subsequent landmark analysis focusing on the TEI demonstrated that, when excluding patients receiving neoadjuvant chemotherapy, upper mediastinal LND may be indicated even when the EIL exceeds 3 cm [15]. Consistent with these previous reports, our univariate analysis revealed that a UGI-EIL of 30 mm or longer was significantly associated with a high incidence of MLN metastasis. In our cohort, right and left RLN node stations (#106recR and #106recL) exhibited high metastatic rates exceeding 12%, which clinically supports the necessity of intensive perioperative treatment and/or upper mediastinal clearance for high-risk longitudinal lesions. However, as mentioned above, preoperatively measuring EIL can be highly subjective and technically difficult in the presence of circumferential strictures or large hiatal hernias. While previous nationwide studies in Japan placed a high clinical emphasis on endoscopic diagnosis and verified its utility using resected specimens [11,14], determining a definitive, reproducible treatment strategy based solely on mucosal longitudinal measurements remains challenging in daily practice. In contrast, selecting the optimal clinical strategy requires a more objective and consistent preoperative assessment modality. From this viewpoint, evaluating EIL via the UGI series serves as a highly objective and robust tool that can be constantly reproduced across institutions, except in cases with complete luminal obstruction. Given that a UGI-EIL ≥ 30 mm was significantly associated with MLN metastasis in our cohort, it represents a highly valuable clinical surrogate. To further enhance diagnostic accuracy, novel imaging modalities, such as the recently reported evaluation of EIL utilizing PET-CT [28], warrant continuous investigation in future clinical trials.

Interestingly, our multivariate model demonstrated that morphological changes in the lymph nodes themselves (MLN size ≥ 5 mm on CT) and deep local tumor invasion (cT3 or deeper) were also powerful independent preoperative predictors of MLN metastasis. This strongly implies that clinical tumor depth is a crucial driver of MLN involvement, reflecting an aggressive biological phenotype capable of vertical lymphatic permeation into the rich esophageal submucosal network. These findings underscore the importance of predicting MLN metastasis by taking into account not only EIL but also the estimated tumor depth. Indeed, the combination of both parameters successfully allowed us to stratify the risk of MLN metastasis within our study cohort.

Historically, diagnosing lymph-node metastasis based on size criteria via conventional CT has been challenging and inconsistent [16,17,18]. For instance, a seminal prospective trial in Japan defined clinically positive lymph nodes as having a short-axis diameter of 8 mm or larger on CT, or exhibiting positive FDG uptake on PET-CT [15]. In contrast, our ROC analysis identified a smaller threshold of 5 mm as the optimal cut-off value for MLN metastasis, achieving an excellent sensitivity of 90.5% and a specificity of 92.3% for histological positivity. This lower threshold indicates that even slightly enlarged mediastinal nodes should be treated with high suspicion in EGJAC, given the dense, multidirectional lymphatic drainage networks surrounding the esophagogastric junction.

In the subgroup analysis for Siewert Type I tumors, cT3 or deeper, MLN size ≥ 5 mm on CT, and FDG uptake on PET-CT were significant indicators for preoperatively predicting MLN metastasis, as well as the entire cohort. Conversely, UGI EIL ≥ 30 mm was not identified as a significant factor. This lack of significance is likely because 41 out of the 44 Siewert Type I cases (93.2%) included in this study already presented with a UGI EIL of >30 mm. In cases with long EILs, such as Siewert Type I tumors, it might be necessary to determine the treatment strategy by taking endoscopic findings into consideration.

Given these discrepancies, optimal preoperative diagnostic criteria for MLN metastasis remain controversial and clearly warrant further extensive investigation. Combining a clinical tumor depth of cT3 or deeper, a 5 mm MLN size threshold, and a UGI EIL ≥ 30 mm seems to provide clinicians with highly objective, reproducible, and easily accessible criteria during initial staging. In patients with both UGI EIL ≥ 30 mm and a cT3 or deeper tumor, the rate of MLN metastasis was exceedingly high, suggesting that MLN metastasis should be considered even when the MLN size is less than 5 mm. Conversely, in patients who did not meet both criteria, no MLN metastasis was observed in the present study, indicating that intensive perioperative treatment and MLND may be omitted. For patients presenting with either UGI EIL ≥ 30 mm or cT3 or deeper, the therapeutic approach should be determined on an individual basis depending on the patient’s condition; however, in our cohort, all cases with positive MLN within this specific subgroup had an MLN size ≥ 5 mm. Therefore, a treatment strategy incorporating MLN size should be considered.

Several limitations must be acknowledged in this study. First, the retrospective nature of the study inherently introduces the potential for selection bias and confounding factors that are unavoidable in non-randomized designs. Throughout the study period, our institution selected surgical procedures in accordance with the aforementioned surgical algorithm. However, for cases with cT1/2 and EIL of 2–3 cm, upper MLND was omitted, which may have led to an underestimation of MLN metastasis in this subgroup. Nevertheless, no local recurrence was observed in these patients, suggesting that the likelihood of histologically positive MLNs was extremely low. Second, the direct therapeutic impact of extended mediastinal LND on survival outcomes was not fully evaluated in this analysis. However, because our survival data clearly confirmed that patients with positive MLN metastasis had a significantly worse prognosis, predicting the presence of occult MLN metastasis is of paramount clinical importance for deciding the optimal perioperative treatment strategy, such as the administration of intensive neoadjuvant therapies. To further elucidate the survival benefits of specific nodal stations, we plan to evaluate the station-specific TEI in a future validation study. Third, the proportion of patients who received neoadjuvant chemotherapy (NAC) was relatively low (19.5%) in our current cohort. Currently, the global standard of care for advanced EGJAC differs substantially between Western countries and Japan. In Western nations, perioperative chemotherapy using the FLOT regimen [3] or neoadjuvant chemoradiotherapy (NACRT) using the CROSS regimen [4] is standard, and the effectiveness of both remains under debate [5]. Further, the recent results of the MATTERHORN trial have demonstrated the prominent efficacy of adding durvalumab to the FLOT regimen (d-FLOT), making perioperative chemoimmunotherapy a new standard of care worldwide [29]. Conversely, in Japan, upfront surgery followed by adjuvant chemotherapy has historically been preferred, although the ongoing NEO-JPEG trial is currently evaluating the utility of intensive neoadjuvant strategies for EGJAC [30]. It should be emphasized that our study focuses primarily on the prediction and detection of MLN metastasis rather than evaluating surgical efficacy. Although the optimal approach and extent of MLND in patients receiving these potent neoadjuvant therapies must be re-evaluated in future multi-institutional collaborative studies with larger sample sizes, our objective preoperative indices for predicting MLN metastasis will remain pivotal for identifying the ideal candidates for such intensive multimodal treatment algorithms.

## 5. Conclusions

In conclusion, our study underscores that pStage and MLN metastasis are critical determinants of long-term survival in Siewert Type I and II EGJAC. EIL, clinical tumor depth, and the size of MLN serve as powerful, objective, and independent risk factors for MLN metastasis. Utilizing these objective preoperative predictors in combination can assist surgical oncologists in identifying high-risk patients who may be candidates for intensive perioperative treatments and/or extended MLND, ultimately moving toward optimized, personalized therapeutic strategies for EGJAC.

## Figures and Tables

**Table 1:** Patient background data.

Variables	Value (*n* = 133)
Age (years)	67.0 (41.0–85.0)
Sex	
Male	127 (95.5%)
Female	6 (4.5%)
BMI (kg/m^2^)	22.8 (16.0–35.1)
Siewert type	
Type I	44 (33.1%)
Type II	89 (66.9%)
Endoscopic EIL (mm)	30.0 (0.0–110.0)
UGI EIL (mm)	35.0 (0.0–114.0)
cT (UICC 8th)	
1	43 (32.3%)
2	26 (19.5%)
3	53 (39.8%)
4	11 (8.3%)
cN (UICC 8th)	
0	73 (54.9%)
1	29 (21.8%)
2	26 (19.5%)
3	5 (3.8%)
cM (UICC 8th)	
0	125 (94.0%)
1	8 (6.0%)
Clinical stage (UICC 8th)	
I	38 (28.6%)
II	24 (18.0%)
III	38 (28.6%)
IV	33 (24.8%)
Preoperative treatment	
No	107 (80.5%)
Yes	26 (19.5%)

BMI, body mass index; EIL, esophageal involvement length; UGI, upper gastrointestinal series.

**Table 2:** Surgical outcomes.

Variable	Value (N = 133)
Surgical Procedure	
McKeown esophagectomy	76 (57.1%)
Ivor Lewis esophagectomy	25 (18.8%)
Proximal gastrectomy	30 (22.6%)
Total gastrectomy	2 (1.5%)
Minimally Invasive Surgery	95 (71.4%)
Lymphadenectomy	
Upper mediastinal	77 (57.9%)
Middle mediastinal	97 (72.9%)
Lower mediastinal	126 (94.7%)
Abdominal	132 (99.2%)
Operative Time (min)	488 (150–838)
Blood Loss (mL)	402 (36–3895)
Postoperative Complications	
Overall morbidity	30 (22.6%)
Pneumonia	4 (3.0%)
RLN paralysis	0
Anastomotic leakage	18 (13.5%)
SSI	5 (3.8)
Others	3 (2.3%)
pT (UICC 8th)	
0	4 (3.0%)
1	52 (39.1%)
2	18 (13.5%)
3	52 (39.1%)
4	7 (5.3%)
pN (UICC 8th)	
0	61 (45.9%)
1	24 (18.0%)
2	21 (15.8%)
3	27 (20.3%)
pM (UICC 8th)	
0	125 (94.0%)
1	8 (6.0%)
pStage (UICC 8th)	
0	2 (1.5%)
I	44 (33.1%)
II	21 (15.8%)
III	36 (27.1%)
IV	30 (22.6%)
MLN Metastasis	42 (31.6%)
Upper	21 (15.8%)
Middle	16 (12.0%)
Lower	31 (23.3%)

RLN, recurrent laryngeal nerve; SSI, surgical site infection; MLN, mediastinal lymph node.

**Table 3:** Details of mediastinal lymph-node metastasis.

Station Number	Station Name	Metastasis, n (%)
No. 105	Cervical paraesophageal	4 (5.2%)
No. 106recL	Left recurrent laryngeal nerve	9 (13.2%)
No. 106recR	Right recurrent laryngeal nerve	9 (12.7%)
No. 106tbL	Left tracheobronchial	4 (7.8%)
No. 107	Subcarinal	7 (8.2%)
No. 108	Middle thoracic paraesophageal	11 (11.6%)
No. 109L	Left pulmonary hilar	4 (4.9%)
No. 109R	Right pulmonary hilar	6 (7.4%)
No. 110	Lower thoracic paraesophageal	19 (15.7%)
No. 111	Supradiaphragmatic	6 (6.1%)
No. 112	Posterior mediastinal	11 (12.5%)

**Table 4:** Univariate analysis for preoperative risk factors for mediastinal lymph-node metastasis.

Variable	Category	N	Odds Ratio	95% CI	*p*-Value
Age (years)	≥75 vs. <75	133	1.06	(0.45–2.50)	0.198
Sex	Male vs. Female	133	0.15	(0.01–3.54)	0.091
BMI (kg/m^2^)	≥25 vs. <25	133	1.62	(0.75–3.52)	0.068
Siewert type	Type 1 vs. Type 2	133	2.51	(1.17–5.38)	0.006
Endoscopic EIL	≥60 mm vs. <60 mm	86	10.36	(2.85–37.72)	<0.001
UGI EIL	≥30 mm vs. <30 mm	127	15.68	(4.82–50.98)	<0.001
cT (UICC 8th)	T3/T4 vs. T1/T2	133	8.18	(3.42–19.60)	<0.001
Preoperative therapy	Yes vs. No	133	1.8	(0.75–4.35)	0.072
MLN size	≥5 mm vs. <5 mm	133	96.39	(27.92–332.74)	<0.001
FDG uptake	Positive vs. Negative	99	8.17	(2.19–30.54)	<0.001

BMI, body mass index; EIL, esophageal involvement length; UGI, upper gastrointestinal series; MLN, mediastinal lymph nodes; FDG, fluorodeoxyglucose.

**Table 5:** Multivariate analysis for the preoperative risk factors for mediastinal lymph-node metastasis.

Variable	Category	Adjusted Odds Ratio	95% CI	*p*-Value
Siewert type	Type 1 vs. Type 2	0.64	(0.16–2.48)	0.272
UGI EIL	≥30 mm vs. <30 mm	7.95	(1.48–42.60)	0.008
cT (UICC 8th)	T3/T4 vs. T1/T2	3.61	(0.92–14.19)	0.043
MLN size	≥5 mm vs. <5 mm	50.46	(13.68–186.18)	<0.001

UGI, upper gastrointestinal series; EIL, esophageal involvement length; MLN, mediastinal lymph nodes.

**Table 6:** Univariate analysis for the preoperative risk factors for mediastinal lymph-node metastasis in Siewert Type I tumors.

Variable	Category	N	Odds Ratio	95% CI	*p*-Value
Age (years)	≥75 vs. <75	44	1.68	(0.33–8.54)	0.367
Sex	Male vs. Female	44	0.38	(0.00–36.74)	1
BMI (kg/m^2^)	≥25 vs. <25	43	0.64	(0.16–2.58)	0.282
Endoscopic EIL	≥60 mm vs. <60 mm	30	6.12	(1.19–31.45)	0.01
UGI EIL	≥30 mm vs. <30 mm	43	1.44	(0.13–16.19)	1
cT (UICC 8th)	T3/T4 vs. T1/T2	44	17.27	(3.45–86.52)	<0.001
Preoperative therapy	Yes vs. No	44	2.75	(0.60–12.68)	0.113
MLN size	≥5 mm vs. <5 mm	44	79.86	(10.24–622.63)	<0.001
FDG uptake	Positive vs. Negative	37	6.12	(1.19–31.45)	0.01

BMI, body mass index; EIL, esophageal involvement length; UGI, upper gastrointestinal series; MLN, mediastinal lymph nodes; FDG, fluorodeoxyglucose.

**Table 7:** Risk stratification of mediastinal lymph-node metastasis combining estimated tumor depth and esophageal involvement length.

UGI EIL ≥ 30 mm	cT3 or Deeper	
	Negative	Positive
Negative	0/42 (0%)	3/14 (21.4%)
Positive	8/27 (29.6%)	31/50 (62%)

UGI, upper gastrointestinal series; EIL, esophageal involvement length.

## Data Availability

The raw data supporting the conclusions of this article will be made available by the authors upon request.

## Abbreviations

The following abbreviations are used in this manuscript:

BMI	Body Mass Index
CI	Confidence Interval
CROSS	ChemoRadiotherapy for Esophageal Cancer Received Survival Study
EGJ	Esophagogastric Junction
EGJAC/EGJAD	Esophagogastric Junction Adenocarcinoma
EIL	Esophageal Involvement Length
ESCC	Esophageal Squamous Cell Carcinoma
FDG	Fluorodeoxyglucose
FLOT	Fluorouracil, Leucovorin, Oxaliplatin, and Docetaxel
GI	Gastrointestinal
JES	Japan Esophageal Society
JGCA	Japanese Gastric Cancer Association
LND	Lymph-Node Dissection
MLN	Mediastinal Lymph Node
MLND	Mediastinal Lymph-Node Dissection
NAC	Neoadjuvant Chemotherapy
NACRT	Neoadjuvant Chemoradiotherapy
OR	Odds Ratio
OS	Overall Survival
RLN	Recurrent Laryngeal Nerve
ROC	Receiver Operating Characteristic
TEI	Therapeutic Efficacy Index/Therapeutic Elongation Index
TNM	Tumor, Node, Metastasis
UGI	Upper Gastrointestinal
UICC	Union for International Cancer Control
